# The use of remimazolam *versus* propofol for induction and maintenance of general anesthesia: A systematic review and meta-analysis

**DOI:** 10.3389/fphar.2023.1101728

**Published:** 2023-02-06

**Authors:** Ching-Chung Ko, Kuo-Chuan Hung, Amina M. Illias, Chong-Chi Chiu, Chia-Hung Yu, Chien-Ming Lin, I-Wen Chen, Cheuk-Kwan Sun

**Affiliations:** ^1^ Department of Medical Imaging, Chi Mei Medical Center, Tainan city, Taiwan; ^2^ Department of Health and Nutrition, Chia Nan University of Pharmacy and Science, Tainan city, Taiwan; ^3^ Department of Anesthesiology, Chi Mei Medical Center, Tainan, Taiwan; ^4^ Department of Anesthesiology, Chang Gung Memorial Hospital, Taoyuan, Taiwan; ^5^ Graduate Institute of Clinical Medical Sciences, College of Medicine, Chang Gung University, Taoyuan, Taiwan; ^6^ Department of General Surgery, E-Da Cancer Hospital, I-Shou University Kaohsiung city, Kaohsiung City, Taiwan; ^7^ School of Medicine for International Students, College of Medicine, I-Shou University, Kaohsiung City, Taiwan; ^8^ Department of Medical Education and Research, E-Da Cancer Hospital, Kaohsiung city, Taiwan; ^9^ Department of Anesthesiology, Chi Mei Medical Center, Liouying, Tainan city, Taiwan; ^10^ Department of Emergency Medicine, E-Da Hospital, I-Shou University, Kaohsiung City, Taiwan

**Keywords:** anesthesia, remimazolam, hypotension, propofol, general anesthesia (GA)

## Abstract

**Background:** The primary objective of this study was to compare the risk of hypotension, as well as the induction and recovery characteristics between remimazolam and propofol in patients receiving surgery under general anesthesia.

**Methods:** The Embase, Medline, Google scholar, and the Cochrane Library databases were searched from inception to March 2022 for randomized controlled trials The primary outcome was the risk of post-induction hypotension between the two agents, while the secondary outcomes included anesthetic depth, induction efficacy, time to loss of consciousness (LOC), hemodynamic profiles, time to eye opening, extubation time as well as the incidence of injection pain and postoperative nausea/vomiting (PONV).

**Results:** Meta-analysis of eight studies published from 2020 to 2022 involving 738 patients revealed a significantly lower risk of post-induction hypotension with the use of remimazolam compared to that with propofol [risk ratio (RR) = 0.57, 95% confidence interval (CI): 0.43 to 0.75, *p* < 0.0001, I^2^ = 12%, five studies, 564 patients]. After anesthetic induction, the anesthetic depth measured by bispectral index (BIS) was lighter in the remimazolam group than that in the propofol group (MD = 9.26, 95% confidence interval: 3.06 to 15.47, *p* = 0.003, I^2^ = 94%, five studies, 490 patients). The time to loss of consciousness was also longer in the former compared to the latter (MD = 15.49 s, 95%CI: 6.53 to 24.46, *p* = 0.0007, I^2^ = 61%, three studies, 331 patients). However, the use of remimazolam correlated with a lower risk of injection pain (RR = 0.03, 95%CI: 0.01 to 0.16, *p* < 0.0001, I^2^ = 0%, three studies, 407 patients) despite comparable efficacy of anesthetic induction (RR = 0.98, 95%CI: 0.9 to 1.06, *p* = 0.57, I^2^ = 76%, two studies, 319 patients). Our results demonstrated no difference in time to eye opening, extubation time, and risk of PONV between the two groups.

**Conclusion:** Remimazolam was associated with a lower risk of post-induction hypotension after anesthetic induction compared with propofol with similar recovery characteristics. Further studies are required to support our findings.

**Systematic Review Registration:** https://www.crd.york.ac.uk/prospero/; Identifier: CRD42022320658.

## Introduction

Intraoperative and postoperative hypotension have been found to be major contributors to adverse outcomes and subsequent healthcare resource consumption (Walsh et al., 2013; Hallqvist et al., 2016; Löffel et al., 2020; Stapelfeldt et al., 2021). A multicenter retrospective cohort study focusing on 368,222 patients undergoing non-cardiac surgical procedures reported a correlation between intraoperative hypotension and an increased incidence of 30-day major adverse cardiac or cerebrovascular events that is magnified with increasing hypotension severity (Gregory et al., 2021). Compared with intraoperative hypotension, post-induction hypotension (i.e., before surgical incision) can also increase the risk of prolonged postoperative stay and/or death (Reich et al., 2005). Another study that investigated the incidence of postoperative kidney injury in 42,825 patients who underwent elective non-cardiac surgery demonstrated a significant association of the risk of acute kidney injury with the depth and duration of hypotension, both before (i.e., post-induction) and after surgical incision (Maheshwari et al., 2018). Accordingly, a number of studies have attempted to identify the risk factors for hypotension after anesthetic induction (Südfeld et al., 2017; Jor et al., 2018; Siriopol et al., 2021; Sutthibenjakul and Chatmongkolchart, 2021; Tarao et al., 2021). Taking into account the high incidence of hypotension after induction of anesthesia (Reich et al., 2005; Jor et al., 2018; Khan et al., 2020; Tarao et al., 2021), there is a serious concern over its prevention by modifying the pharmacological strategies. Although propofol is one of the most popular drugs for sedation and anesthesia in clinical practice because of its characteristics of rapid action time and prompt recovery (Elbakry et al., 2018; Zhang et al., 2018), it has been reported to be associated with adverse effects such as pain at the injection site, hemodynamic instability, and dose-related respiratory depression (Ho et al., 1999; Ebert, 2005; Chen et al., 2021a; Sneyd et al., 2022). The mechanisms of propofol-associated decrease in mean blood pressure (MBP) include a diminished sympathetic tone that results in vasodilation and a reduction in total peripheral resistance (Sahinovic et al., 2018). Pooled evidence revealed that the use of propofol for anesthetic induction remains a significant contributor to hypotension (Chen et al., 2021b).

Remimazolam, which is an ester-based benzodiazepine, is a high-affinity and selective ligand for the benzodiazepine site on the gamma-aminobutyric acid (GABA)_A_ receptor and is rapidly hydrolyzed to an inactive metabolite by tissue esterases (Kilpatrick et al., 2007). Previous studies showed that remimazolam has a predictable duration of action and allows rapid recovery when being used in sedation procedures (e.g., gastrointestinal endoscopy) (Borkett et al., 2015; Pambianco et al., 2016; Rex et al., 2018; Pastis et al., 2019). For anesthetic induction, continuous intravenous infusion at a dose of 12 mg/kg/h (or cumulative dosage of 0.29 mg/kg) has been reported to attain a loss of consciousness for about 90 s (Doi et al., 2020). Regarding the hemodynamic impact of remimazolam, although a study has reported a lower risk of post-induction hypotension compared to that with propofol in patients receiving cardiac valve surgery (e.g., 43.3%–16.7%) (Liu et al., 2021), there is a lack of pooled evidence through a systematic approach. Therefore, the primary objective of this study was to compare the risk of hypotension between remimazolam and propofol in patients receiving surgery under general anesthesia, while the secondary outcomes included a comparison of induction [i.e., anesthetic depth, induction efficacy, time to loss of consciousness (LOC), and incidence of injection pain], post-induction (i.e., hemodynamic profiles), and recovery [i.e., time to eye opening, extubation time, and postoperative nausea/vomiting (PONV)] characteristics between the two agents.

## Materials and methods

This meta-analysis was conducted in accordance with the recommendations of the PRISMA statement and registered with the International Prospective Register of Systematic Reviews (CRD42022320658).

### Data sources and searches

We searched the Embase, Medline, Google scholar, and the Cochrane Library databases from inception to 24 March 2022, using the following search terms: (“General anesthesia*" or “tracheal intubation” or “anesthetic induction” or “extubation” or “surgery” or “postoperative” or “intraoperative” or “perioperative” or “surgical patients” or “induction”) and (“Remimazolam” or “CNS 7056″) limited to RCTs. There were no restrictions on language, gender, sample size, and geographic location during literature search, which did not include gray literature. Supplementary Table S1 shows the search strategy for one of the databases. We manually searched the Google scholar database to retrieve related articles. Following the identification of a relevant article, the efficiency of literature search was enhanced by adopting a forward snowballing strategy (Greenhalgh and Peacock, 2005; Vassar et al., 2016) to find additional records through reviewing the reference lists of the retrieved studies for potentially eligible articles to be included in the present meta-analysis.

### Inclusion criteria

To assess the eligibility of the acquired studies for the current meta-analysis, we adopted the following criteria: (a) Population: Adult patients (age ≥18 years) receiving surgery under only general anesthesia, (b) Intervention: The administration of remimazolam as an agent for anesthetic induction or maintenance of general anesthesia, (c) Comparison: The use of propofol as an induction agent, (d) Outcomes: Incidence of hypotension (i.e., primary outcome), as well as anesthetic depth after induction, hemodynamic profiles, and other clinical characteristics (e.g., time to LOC, time to eye opening, extubation time, injection pain, induction efficacy, and incidence of PONV) (i.e., secondary outcomes). We only included RCTs for analysis and contacted the authors of the included studies with missing information for availability of the original data.

### Exclusion criteria

Exclusion criteria were: (1) Patients under general anesthesia combined with regional analgesia (e.g., epidural anesthesia) or peripheral nerve block; (2) studies without the use of propofol as the control group; (3) those focusing on patients receiving surgery under sedation; (4) those without information about outcomes, and (5) RCTs not published as full-length original research, namely, those presented as letters, abstracts, reviews, case reports, or other forms of publications.

### Study selection

Two authors independently scrutinized the titles and abstracts of the identified RCTs for eligibility of being included in the current study. The full texts of the potentially eligible studies were independently examined based on their inclusion and exclusion criteria. Discrepancies in opinions about the eligibility of a particular study were resolved through discussion with a third reviewer.

### Data extraction

Items retrieved from each trial included: First author, year of publication, age, gender, body mass index (BMI), American Society of Anesthesiologists Physical Status (ASA-PS), sample size, dosage of remimazolam, type of procedures, efficacy of induction, hemodynamic profiles (e.g., heart rate), extubation time, time to LOC, time to eye opening, depth of anesthesia immediately after anesthetic induction, risk of hypotension as well as the incidence of PONV and injection pain. Disagreements were settled by consulting a third author.

### Definitions

The primary outcome was the risk of hypotension immediately after anesthetic induction between the two agents, while the secondary outcomes included anesthetic depth, induction efficacy, time to LOC, hemodynamic profiles, time to eye opening, extubation time as well as the incidence of injection pain and PONV. The definitions of induction efficacy and hypotension were in accordance with those of each study. If one study did not define the events for hypotension, we considered the use of vasopressor to be an indicator of hypotension. If a study reported outcomes (e.g., hemodynamic profiles or anesthetic depth) at different time points, we only analyzed the data acquired just after anesthetic induction. In addition, the data obtained following tracheal intubation was not used for analysis. As a previous study reported that a remimazolam dosage of 12 mg/kg/h or an accumulative dose of 0.3 mg/kg provided a mean time to LOC of 88.7 s which may be comparable to that offered by propofol (i.e., 78.7 s at a conventional dosage of 2**–**2.5 mg/kg) (Doi et al., 2020), we adopted the data associated with a remimazolam dosage of 12 mg/kg/h for comparison with the propofol group when several doses of remimazolam were reported in one study. Subgroup analysis was performed based on the type of surgery (i.e., cardiac vs. non-cardiac).

### Risks of bias assessment

By employing the Cochrane’s tool (RoB 2), two authors independently assessed the possibility of different biases across the included RCTs, namely, allocation, performance, attrition, measurement, reporting, and overall biases (Sterne et al., 2019). Disagreement between the two authors was resolved through arbitration of a third reviewer.

### Effect measures and data synthesis

The current study used the Cochrane Review Manager (RevMan 5.3; Copenhagen: The Nordic Cochrane Centre, The Cochrane Collaboration, 2014) for data synthesis. Data of an outcome from at least two trials were pooled. Considering potential heterogeneities in clinical setting and dosage of remimazolam across the included studies, we used the Mantel–Haenszel random-effects model for the analysis of dichotomous outcome data and presented the results as risk ratios (RR) with 95% confidence intervals (CIs). For outcomes of continuous variables, we reported their mean differences (MD) and 95% CIs. Using the I^2^ statistic, we predefined significant heterogeneity as I^2^ >50% (Higgins et al., 2003). The potential publication bias for a specific outcome mentioned in 10 or more trials was evaluated through visual inspection of a funnel plot. The potential impact of the findings of each trial on the overall results was determined with “leave-one-out” sensitivity analysis (Hung et al., 2022a; Hung et al., 2022b). For all comparisons, two-tailed tests were conducted with statistical significance being set at a *p*-value under 0.05.

### Certainty assessment

The certainty of the evidence regarding the primary and secondary outcomes according to study limitations, effect consistency, the probability of publication bias as well as indirectness and imprecision included in Grading of Recommendations Assessments, Development, and Evaluation (GRADE) was independently evaluated by two authors who assigned the outcomes of their assessments to one of four grades, ranging from high, moderate, low, to very low. Disagreements regarding certainty ratings were settled through discussion.

## Results

### Search results and study characteristics

**FIGURE 1:**
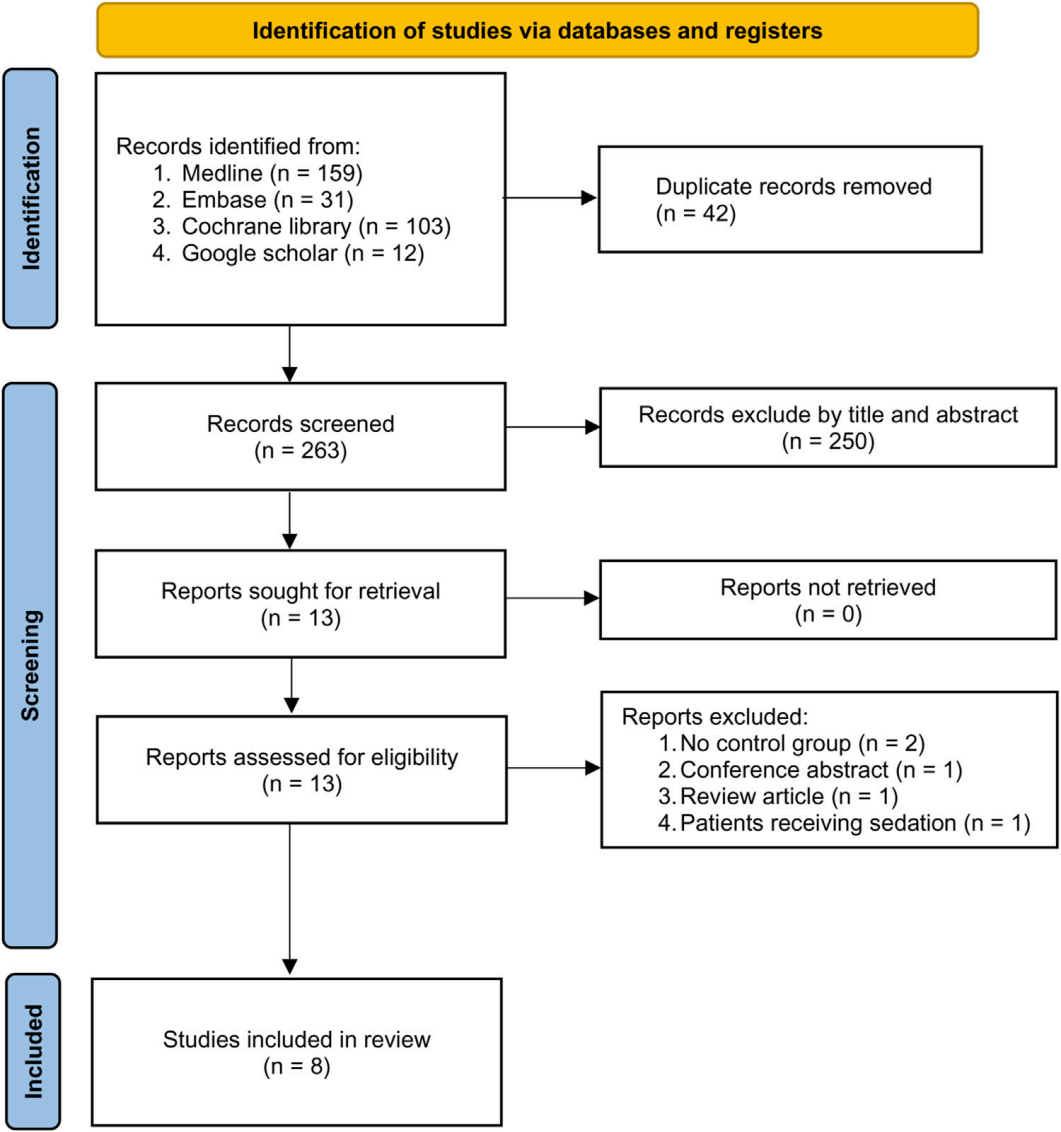
PRISMA flow diagram of study selection for the current meta-analysis.

The process of study selection is depicted in Figure 1. Following the exclusion of 42 duplicates and 250 articles deemed unsuitable according to titles and abstracts from the 305 potentially relevant records retrieved from the databases, 13 potentially eligible studies were subjected to full-text review. After removing five more studies based on our exclusion criteria, eight randomized controlled trials (RCT) published from 2020 to 2022 involving 738 patients were included in the current meta-analysis (Doi et al., 2020; Li et al., 2021a; Dai et al., 2021; Liu et al., 2021; Tang et al., 2021; Hari et al., 2022; Hasegawa et al., 2022; Shi et al., 2022). The characteristics of the included studies are demonstrated in Table 1. All studies enrolled participants of both genders with the prevalence of males ranging from 0% to 56.3%. The mean age of the recruited individuals varied from 35 to 56 years. The sample size of the included studies ranged from 30 to 225. Of the eight studies involving operations under general anesthesia with tracheal intubation, six involved non-cardiac procedures and two focused on valve surgery (Liu et al., 2021; Tang et al., 2021). Four trials provided information regarding surgical time, which was between 27 and 250 min (Doi et al., 2020; Tang et al., 2021; Hari et al., 2022; Shi et al., 2022). Although all studies compared remimazolam with propofol during anesthetic induction, the doses and administration techniques of remimazolam varied. While remimazolam was administered as bolus doses of 0.2 mg/kg and 0.3 mg/kg in two (Li et al., 2021a; Shi et al., 2022) and three (Dai et al., 2021; Liu et al., 2021; Tang et al., 2021) trials, respectively, it was given as continuous infusion at a dosage of 12 mg/kg/h in three studies (Doi et al., 2020; Hari et al., 2022; Hasegawa et al., 2022). The dosages of propofol also varied in the control group with a maximal dosage of 2.5 mg/kg (Table 1). All studies were published in the English language. Of the eight included trials, five were conducted in China (Li et al., 2021a; Dai et al., 2021; Liu et al., 2021; Tang et al., 2021; Shi et al., 2022) and three were reported from Japan (Doi et al., 2020; Hari et al., 2022; Hasegawa et al., 2022).

**TABLE 1:** Characteristics of studies (n = 8).

	Age (year)^a^	Male (%)	BMI (kg/m^2^) or weight (kg)^a^	ASA-PS	N	Procedures	Surgical time (minutes)^a^	Induction dosage (Remi vs. Pro)	Anesthesia maintenance	Country
Dai 2021	48 vs. 52	42.4	25 vs. 25	I-II	99	Mixed surgery	NA	0.3 mg/kg vs. 2 mg/kg	Sevoflurane	China
Doi 2020	56 vs. 56	50.7	23	I-II	225	Mixed surgery	144 vs. 123	12 mg/kg/h vs. 2–2.5 mg/kg	Remi or Pro	Japan
Hari 2022	45 vs. 47	0	59 vs. 61	I-III	64	LGS	154 vs. 160	12 mg/kg/h vs. 1–1.5 mg/kg	Remi or Des	Japan
Hasegawa 2022	35 vs. 42	53	64 vs. 59	I-II	30	Mixed surgery	NA	12 mg/kg/h vs. 0.5 mg/kg^b^	Remi or Pro	Japan
Li 2021	49 vs. 47	47.1	60 vs. 60	I-II	104	NA	NA	0.2 mg/kg vs. 2.5 mg/kg	Remi or Pro	China
Liu 2021	55 vs. 51	51.7	59 vs. 65	III	60	Valve surgery	NA	0.3 mg/kg vs. 2.5 ug/ml^c^	Dex and Pro	China
Shi 2022	53 vs. 52	44.7	66 vs. 68	II-III	76	EVL	27 vs. 27	0.2 mg/kg vs. 2 mg/kg	Remi or Pro	China
Tang 2021	55 vs. 53	56.3	24 vs. 24	I-III	80	Valve surgery	250 vs. 246	0.3 mg/kg vs. 1.5 mg/kg	NA	China

^a^ Presented as Remimazolam vs. Propofol; ASA-PS: American Society of Anesthesiologists Physical Status.

^b^ Patients received propofol at 0.5 mg/kg followed by 10 mg/kg/h.

^c^ Patients received target-controlled infusion (TCI) of propofol 2.5 μg/mL.

^‡^laparoscopic cholecystectomy or robotic gynecologic surgery.

LGS: laparoscopic gynecological surgery; EVL: endoscopic variceal ligation; Remi: Remimazolam; Pro: propofol; Dex: dexmedetomidine; Des: desflurane; NA: not available.

### Risk of bias assessment

**FIGURE 2:**
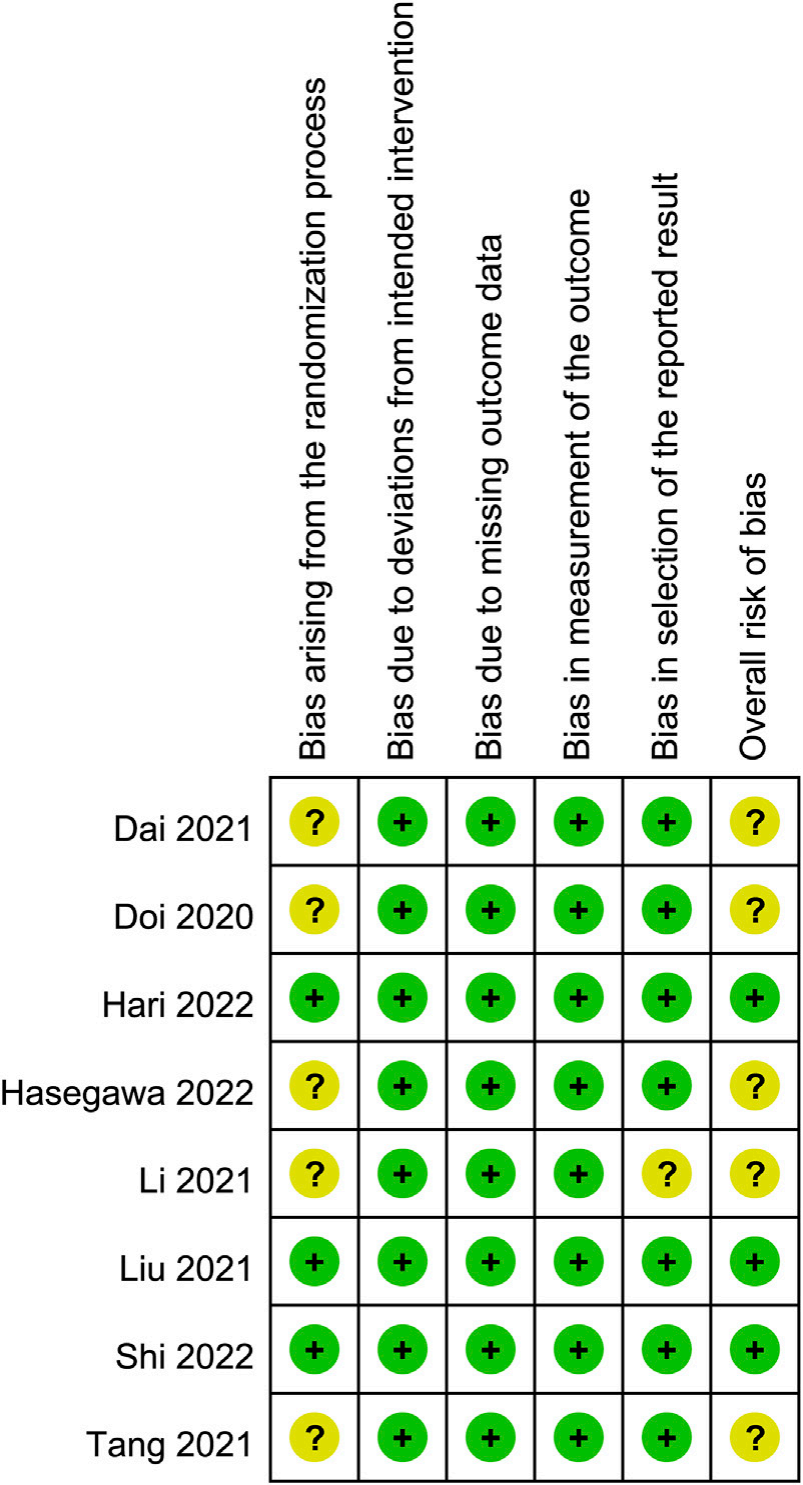
Risks of bias of the included studies. Risk of bias. Green: low risk; yellow: some concern; red: high risk.

The risks of bias of individual studies are presented in Figure 2. In the domain of randomization, five RCTs were considered to be associated with “some concerns” because of a lack of information about allocation concealment. As for the domains of deviation from the intended intervention, missing outcome data, and measurement of outcome, all eight RCTs had a low risk of bias. The domain of selective reporting bias was regarded as having “some concerns” in one trial without information regarding trial registration. As a result, regarding the overall risk of bias, five trials fell into the category of “some concerns” while it was low in the three studies (Figure 2).

### Changes in hemodynamic profiles between remimazolam and propofol

**FIGURE 3:**
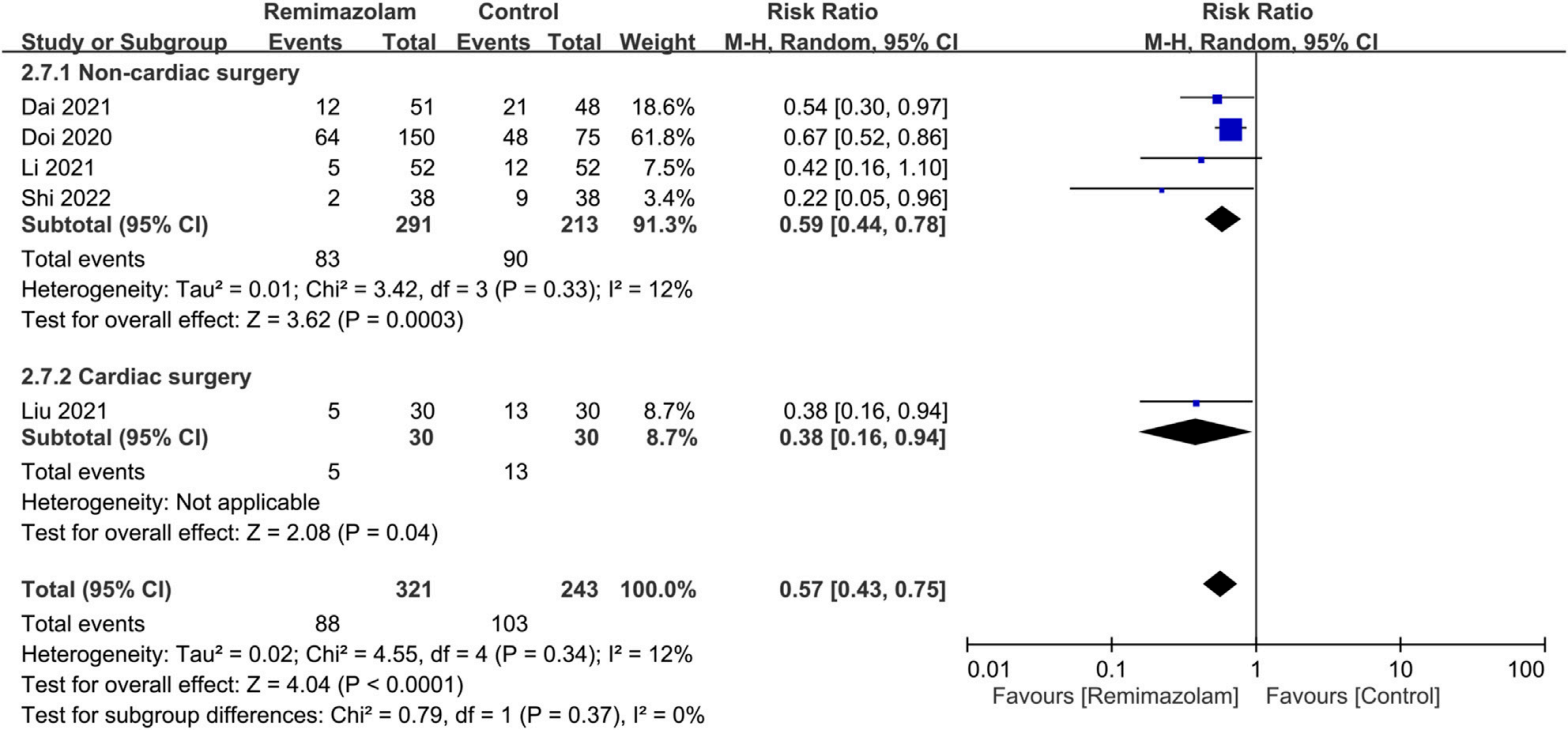
Forest plot comparing the risk of hypotension between remimazolam and control groups. M-H, Mantel-Haenszel; CI, confidence interval.

Our results showed a significantly lower risk of hypotension associated with the use of remimazolam than that with propofol [Risk ratio (RR) = 0.57, 95% CI: 0.43 to 0.75, *p* < 0.0001, I^2^ = 12%, five trials, 564 participants) (Figure 3) (Doi et al., 2020; Li et al., 2021a; Dai et al., 2021; Liu et al., 2021; Shi et al., 2022). Compared with propofol, the use of remimazolam as an induction agent correlated with a higher heart rate [Mean difference (MD) = 4.26 beats/minutes, 95% CI: 0.01 to 8.51, *p* = 0.03, I^2^ = 61%, four trials, 265 participants] (Supplementary Figure S1) (Dai et al., 2021; Liu et al., 2021; Hasegawa et al., 2022; Shi et al., 2022). However, there was no difference in MBP between both groups (MD = 5.79 mmHg, 95% CI: −0.5 to 12.07, *p* = 0.07, I^2^ = 87%, four trials, 265 participants) (Supplementary Figure S2) (Dai et al., 2021; Liu et al., 2021; Hasegawa et al., 2022; Shi et al., 2022). Although subgroup analysis revealed significant impacts of surgical type on heart rate and MBP, there was no effect of surgical type on the risk of hypotension (Figure 3).

### Characteristics of remimazolam during anesthetic induction

**FIGURE 4:**
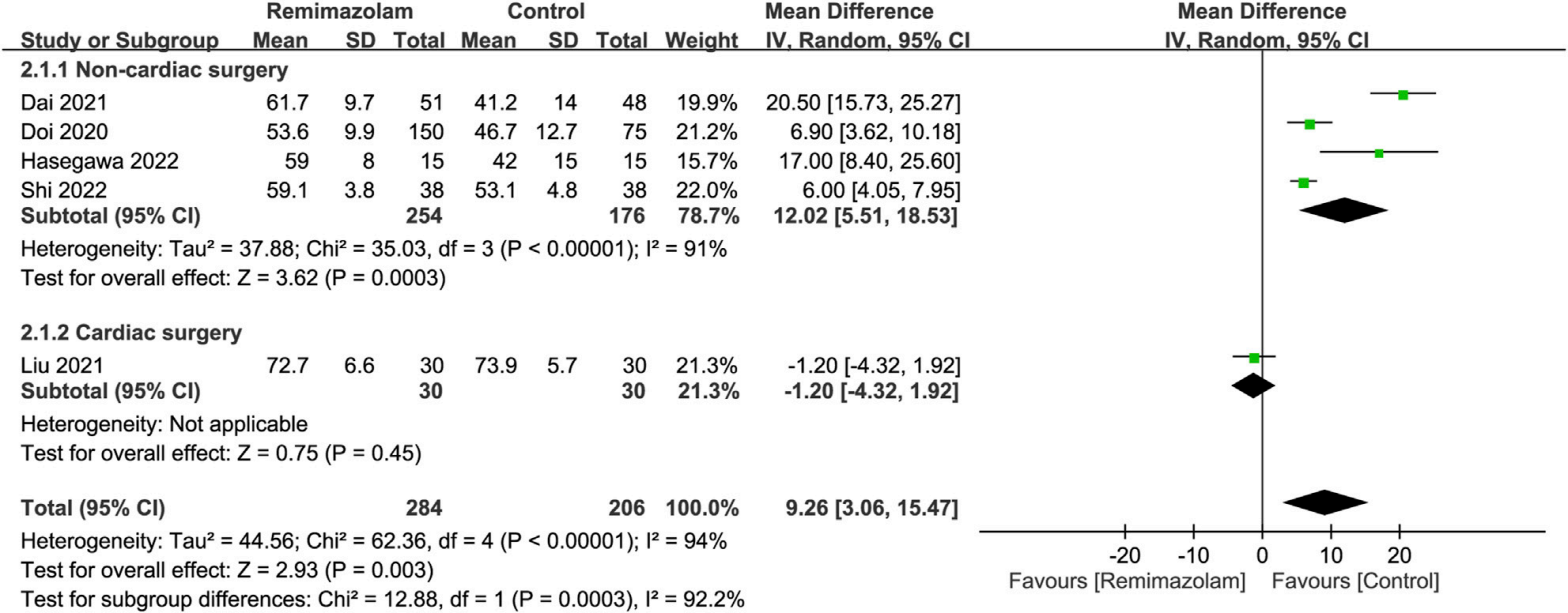
Forest plot comparing anesthetic depth between remimazolam and control groups. CI, confidence interval; IV, inverse variance.

**FIGURE 5:**
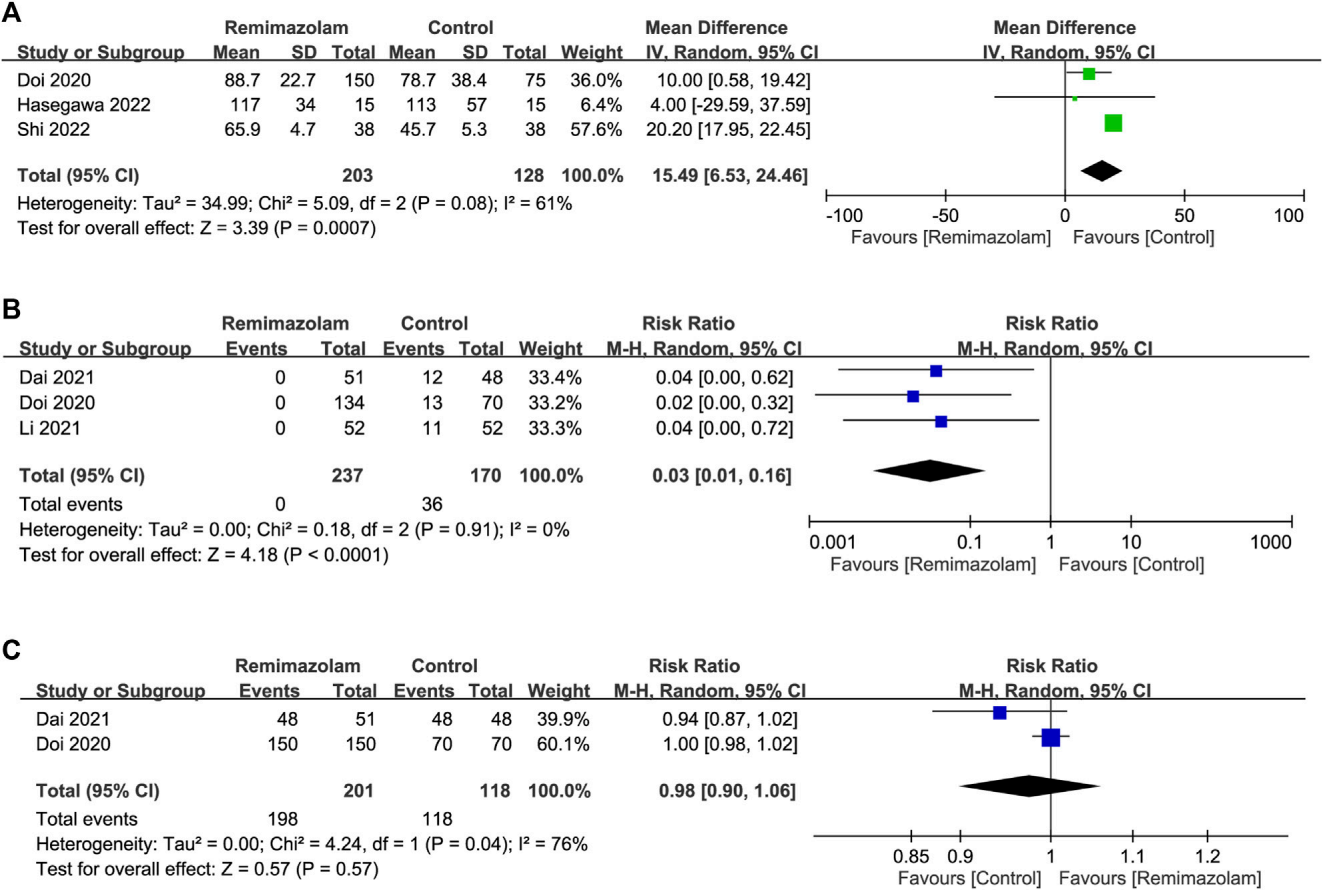
Forest plot comparing **(A)** time to loss of consciousness; **(B)** risk of injection pain and **(C)** efficacy of anesthetic induction between remimazolam and control groups. CI, confidence interval; IV, inverse variance; M-H, Mantel-Haenszel.

All studies investigated the differences in induction characteristics between remimazolam and propofol (Doi et al., 2020; Li et al., 2021a; Dai et al., 2021; Liu et al., 2021; Tang et al., 2021; Hari et al., 2022; Hasegawa et al., 2022; Shi et al., 2022). Of the eight included studies, two provided details regarding the assessment of induction efficacy. While one study defined induction efficacy as the achievement of a combined endpoint comprising a lack of body movements, absence of intraoperative awakening/recall, and no need for rescue sedatives (Doi et al., 2020), it referred to the completion of anesthesia induction without rescue sedation in the other (Dai et al., 2021). After anesthetic induction, the anesthetic depth measured by bispectral index (BIS) was greater in the remimazolam group than that in the propofol group (MD = 9.26, 95% CI: 3.06 to 15.47, *p* = 0.003, I^2^ = 94%, five trials, 490 participants) (Figure 4) (Doi et al., 2020; Dai et al., 2021; Liu et al., 2021; Hasegawa et al., 2022; Shi et al., 2022). The time to LOC was also longer in the former compared to the latter (MD = 15.49 s, 95% CI: 6.53 to 24.46, *p* = 0.0007, I^2^ = 61%, three trials, 331 participants) (Figure 5A) (Doi et al., 2020; Hasegawa et al., 2022; Shi et al., 2022). On the other hand, the use of remimazolam was associated with a lower risk of injection pain compared to propofol (RR = 0.03, 95% CI: 0.01 to 0.16, *p* < 0.0001, I^2^ = 0%, three trials, 407 participants) (Figure 5B) (Doi et al., 2020; Li et al., 2021a; Dai et al., 2021) with comparable efficacy of anesthetic induction between the two agents (RR = 0.98, 95% CI: 0.9 to 1.06, *p* = 0.57, I^2^ = 76%, two trials, 319 participants) (Figure 5C) (Doi et al., 2020; Dai et al., 2021). Subgroup analysis revealed a significantly deeper anesthesia associated with propofol than that with remimazolam in patients undergoing non-cardiac surgery (Figure 4). However, subgroup analysis was not performed on other outcomes as only non-cardiac surgery was involved.

### Recovery characteristics between remimazolam and propofol

Information regarding time to eye opening (Doi et al., 2020; Li et al., 2021a; Shi et al., 2022) and extubation time (Doi et al., 2020; Li et al., 2021a; Tang et al., 2021; Shi et al., 2022) was available in three and four trials, respectively. Three trials adopted remimazolam and propofol to maintain anesthetic depth (Doi et al., 2020; Li et al., 2021a; Shi et al., 2022), while one study involving cardiac surgery did not specify the intraoperative anesthetic agents (Tang et al., 2021). Our results demonstrated no difference in extubation time (MD = −4.59 min, 95% CI -12.31 to 3.13, *p* = 0.24; I^2^ = 98%; four RCTs; n = 485) (Supplementary Figure S3A) (Doi et al., 2020; Li et al., 2021a; Tang et al., 2021; Shi et al., 2022) and time to eye opening (MD = −1.12 min, 95% CI -7.38 to 5.14, *p* = 0.73; I^2^ = 100%; three RCTs; n = 405) (Supplementary Figure S3B) (Doi et al., 2020; Li et al., 2021a; Shi et al., 2022) between the two groups. The incidence of PONV was available in five trials (Li et al., 2021a; Dai et al., 2021; Tang et al., 2021; Hari et al., 2022; Shi et al., 2022), which demonstrated comparable risk of PONV in both groups (RR = 0.82, 95% CI: 0.26 to 2.6, *p* = 0.74, I^2^ = 0%, five trials, 419 participants) (Supplementary Figure S4). While subgroup analysis showed no significant impact of the type of surgery on extubation time and PONV, it was not performed on time to eye opening as only trials of non-cardiac surgery were available.

### Sensitivity analysis

Sensitivity analysis confirmed the robustness of results including the impact of remimazolam on induction efficacy, anesthetic depth, time to LOC and eye opening as well as the risks of injection pain, hypotension, and PONV. However, the outcomes on hemodynamic profiles (i.e., heart rate and MBP) and extubation time were not stable on sensitivity analysis.

### Certainty of evidence

Supplementary Table S2 summarizes the quality of evidence for outcome assessment based on the GRADE system. While the levels of evidence were graded as low for most outcomes due to a high degree of inconsistency and imprecision, they were graded as high in three outcomes (i.e., risk of hypotension, injection pain, and risk of PONV).

## Discussion

Although two review articles (Sneyd and Rigby-Jones, 2020; Kim, 2022) suggested that a principal reason for considering remimazolam as an induction agent of general anesthesia may be its superior hemodynamic stability (Sneyd and Rigby-Jones, 2020; Kim, 2022), clinical experience with remimazolam only comprises a limited number of clinical investigations. This is the first meta-analysis designed to examine the differences in the risk of hypotension as well as induction and recovery characteristics between remimazolam and propofol in the anesthesia setting. Our findings showed that the use of remimazolam was associated with a lower risk of hypotension after anesthetic induction (remimazolam: 27.4% vs. propofol: 42.4%, RR = 0.57). Although propofol may achieve a shorter time to LOC (i.e., 15.49 s) and lower BIS values compared to remimazolam, the efficacy of anesthetic induction was comparable between the two agents with the risk of injection pain being lower in the latter. There was no difference in time to eye opening, extubation, and risk of PONV between patients receiving remimazolam and those being given propofol in the anesthesia setting.

Intraoperative and postoperative hypotension have been recently identified as major risk factors for adverse outcomes in high-risk patients as a short (>5 min) drop in baseline systolic blood pressure by 41–50 mmHg has been reported to correlate with an over three-fold increase in the odds of myocardial infarction (i.e., odds ratio: 3.42) (Hallqvist et al., 2021). Regarding the impact of intraoperative hypotension on post-surgery 30-day risks of major adverse cardiac or cerebrovascular events in the general population, a multicenter retrospective cohort study on over 368 thousand non-cardiac surgical patients showed elevations in the estimated odds by 12%, 17%, and 26% for MBPs of ≤75, ≤65, and ≤55 mmHg, respectively (Gregory et al., 2021). The risk of associated injuries and mortality has also been found to soar with a longer exposure and lower pressure (Wesselink et al., 2018). While a threshold of 5 minutes with systolic BP < 90 mmHg has been reported to be associated with the occurrence of myocardial and renal injury (Ahuja et al., 2020), a hypotensive period of MBPs below 80 mmHg for 10 minutes or more is related to an increased mortality (Wesselink et al., 2018). This raises a serious concern as a previous study demonstrated that up to 9% of patients could experience severe hypotension for 0–10 min after induction of general anesthesia (Reich et al., 2005). A number of predictors of hypotension following anesthetic induction have been reported, including ASA-PS III–V, baseline MBP <70 mmHg, age ≥50 years, increasing induction dosage of fentanyl, and the use of propofol for anesthesia induction (Reich et al., 2005). Indeed, a previous study showed that the use of propofol may lead to a reduction in MBP by 26% after administration (Tsuchiya et al., 2010), highlighting the need for alternative agents in high risk patients.

Remimazolam, which is a full agonist acting at the benzodiazepine binding site of the GABA_A_ receptor (Saari et al., 2011), is subjected to organ-independent hydroxylation by tissue esterases to an inactive metabolite that is rapidly removed from the body (i.e., high clearance) even after prolonged infusions (Antonik et al., 2012; Wesolowski et al., 2016) (Schnider and Minto, 2013). Pharmacokinetically, it has a small volume of distribution and a stable half-life of 6–7 min regardless of the duration of administration (Upton et al., 2010; Schnider and Minto, 2013). Besides, gender- and ethnic-related pharmacokinetic impacts are clinically insignificant (Zhou et al., 2021). Because of these properties, remimazolam is considered an ultra-short-acting anesthetic characterized by rapid onset and recovery (Schüttler et al., 2020). For anesthetic sedation, the use of a single dose from 0.1 mg/kg to 0.2 mg/kg or administration of an initial dose of 2.5–8 mg followed by a top-up dose of 1.25–3 mg is common (Zhu et al., 2021). A previous meta-analysis of two RCTs enrolling 762 patients revealed inferior sedative efficacies but superior hemodynamic profiles of remimazolam to those of propofol in the sedation setting (Zhu et al., 2021). By comparison, the risk of hypotension, efficacy, and recovery profiles associated with a relatively high dose of remimazolam during routine practice in the anesthetic induction setting (i.e., 6–12 mg/kg/h or 0.2–0.3 mg/kg being commonly used (Doi et al., 2020; Dai et al., 2021; Liu et al., 2021; Tang et al., 2021; Hari et al., 2022; Hasegawa et al., 2022; Shi et al., 2022) have not been addressed.

In the current meta-analysis, we found a lower risk of hypotension after anesthetic induction (RR = 0.57) in patients receiving remimazolam compared to those being administered propofol with comparable induction efficacy between the two groups, suggesting the feasibility of remimazolam use in the anesthesia setting especially for those with a high cardiovascular risk. Our demonstration of a low risk of hypotension in patients undergoing non-cardiac and cardiac surgery also supported the safety of remimazolam use in patients with a high risk of hypotension. Despite the encouraging finding, a previous study focusing on 20 elderly patients with severe aortic stenosis reported an incidence of hypotension as high as 70% following intravenous remimazolam at 6 mg/kg/h (Nakanishi et al., 2021). In contrast, although the incidence of hypotension was relatively low at 27.4% (i.e., 88/321, five studies) among patients receiving remimazolam in the current meta-analysis, the high risk indicates the need for its judicious use in critically ill patients.

Compared to propofol, the present meta-analysis showed a higher BIS (MD = 9.26) and a longer time to LOC (MD = 15.49 s) associated with remimazolam use. Despite optimization of the BIS algorithm to give an approximately monotonic and linear response to different doses of propofol or inhalational anesthetics, a previous review has reported that such calibration may not be justified for benzodiazepines (Sneyd and Rigby-Jones, 2020). For instance, the value of BIS for assessing hypnosis in those receiving remimazolam is not as well-defined as for propofol (Sneyd and Rigby-Jones, 2020; Kim, 2022). Therefore, evaluation of remimazolam-related risk of hypotension based on the depth of anesthesia assessed by BIS may not be appropriate. Nevertheless, these induction characteristics of remimazolam may render it unsuitable for use in rapid sequence induction as prolonged LOC may elevate the risk of recall during airway manipulation. Further studies are warranted to address this issue.

Despite the concept of “fast-track anesthesia” in ambulatory surgery to allow early discharge of patients from the operation theater by using short-acting anesthetics, one of the major obstacles was PONV that prolonged post-anesthesia care unit (PACU) stay (Çaparlar et al., 2017), increased workload of PACU staff, and consumed healthcare resources (Belcher et al., 2017). In this aspect, propofol has gained much popularity in clinical practice (Çaparlar et al., 2017) because of its advantage of causing less PONV compared with other agents (Sampson et al., 1988). In the present meta-analysis, our finding of no difference in the risk of PONV in patients receiving remimazolam or propofol is consistent with that of a previous meta-analysis that showed similar risk of PONV between the two agents in the sedation setting (Zhu et al., 2021), supporting the choice of remimazolam as an alternative to propofol.

In the anesthesia setting, the keys to success for ambulatory surgery include the maintenance of stable patient hemodynamics and minimization of untoward side effects as well as ensuring rapid patient emergence and readiness for home discharge (Davis et al., 2000). Compared with midazolam, remimazolam has been found to be associated with a shorter time to recovery and a better cognitive recovery in the sedation setting (Jhuang et al., 2021). When compared to propofol for procedural sedation, a previous meta-analysis showed a lower risk of cardiopulmonary adverse events (e.g., hypotension and hypoxemia) related to remimazolam use (Zhu et al., 2021). The current study further revealed a lower risk of hypotension with remimazolam compared to propofol in spite of similar time to eye opening and extubation as well as the risk of PONV in the anesthesia setting. Therefore, compared to other anesthetic agents (i.e., propofol or midazolam), our findings suggested some desirable properties of remimazolam that may favor its use in the ambulatory surgery setting. Consistently, a previous study that focused on the comparison of anesthesia efficacy between remimazolam and propofol in patients undergoing colonoscopy showed less injection pain, higher patient satisfaction scores, and a lower hypotension risk in those receiving remimazolam group compared to those getting propofol despite the comparable discharge time between the two groups (Yao et al., 2022). The results, therefore, may further support the feasibility of remimazolam use in an ambulatory setting (Yao et al., 2022). Nevertheless, further studies are required to investigate the impact of remimazolam on the time to ambulation, patient satisfaction, and patient readiness for home discharge.

There were several limitations in the current meta-analysis. First, heterogeneity in the dosage of remimazolam, administration technique (i.e., bolus dose or infusion) and strategy (e.g., combined use of opioid) as well as ASA-PS across the included studies remained high in the current meta-analysis. For instance, the limited number of available trials for subgroup analysis of the effect of remimazolam dosage precluded any valuable suggestions regarding dose selection of remimazolam for anesthetic induction. Nevertheless, a previous study reported no difference in efficacy when comparing two induction doses of remimazolam (6 and 12 mg/kg/h) with propofol (2.0–2.5 mg/kg) as a sedative for general anesthesia (Doi et al., 2020). Second, despite a lower risk of hypoxemia associated with remimazolam use compared to that with propofol in the sedation setting as suggested by pooled evidence (Zhu et al., 2021), we were unable to analyze the risk of respiratory depression following surgery between the two agents because of a lack of relevant information. Third, the recruitment of only Asian populations (i.e., Japan and China) may limit the applicability of our findings to patients of different ethnic or geographical backgrounds. Although the inclusion of gray literature may introduce diversity to our study population, questionable authenticity and reliability remain an important concern. Besides, despite the belief that excluding gray literature may result in an overestimation of treatment effects, current research has demonstrated that it only affects a minority of reviews (Schmucker et al., 2017). Fourth, although postoperative prognosis may be an indicator for evaluating the benefit of remimazolam, four out of our eight included studies enrolled relatively healthy participants (i.e., ASA-PS I-II) and three recruited patients with a mixed health status (ASA-PS I-III) while only one trial included those with ASA-PS III. Therefore, assessment of the prognostic impact of anesthetics for induction (i.e., remimazolam vs. propofol) on such a mixed patient population may not be reliable. Indeed, none of the included studies provided relevant information for analysis despite availability of information about the incidence of anesthetic-related postoperative complications (e.g., nausea, vomiting) in four studies. Fifth, despite the variation in the definition of hypotension across the included trials, the result of current meta-analysis showed low heterogeneity (i.e., I^2^ = 12%) that suggested no significant impact of the definition of hypotension on our study outcome. Finally, the relatively small sample size highlighted the preliminary nature of the present study that warrants further large-scale trials to verify its findings in terms of safety, efficacy, and recovery characteristics of remimazolam in the anesthesia setting.

## Conclusion

Despite the demonstration of a benefit of remimazolam over propofol reflected by a lower risk of post-induction hypotension with comparable recovery characteristics (i.e., extubation time, time to eye opening, and risk of PONV), the evidence generated from the current meta-analysis might not be strong enough to support the use of remimazolam over propofol in routine anesthesia practice. Further studies are warranted to compare the two agents focusing on other aspects in the entire anesthesia process.

## Data Availability

The original contributions presented in the study are included in the article/Supplementary Material, further inquiries can be directed to the corresponding author.
