# Supplementary material for: The use of remimazolam versus propofol for induction and maintenance of general anesthesia: A systematic review and meta-analysis
Source: Front Pharmacol. 2023 Feb 6;14:1101728. doi: 10.3389/fphar.2023.1101728 (PMC9939642; doi:10.3389/fphar.2023.1101728)

**Appendix**

**Supplemental Table 1.** Search strategies for Medline

**Supplemental table 2:** Summary of findings for the main comparison

**Supplemental figure 1.** Forest plot comparing heart rate between remimazolam and control groups. CI, confidence interval; IV, inverse variance.

**Supplemental figure 2.** Forest plot comparing mean blood pressure between remimazolam and control groups. CI, confidence interval; IV, inverse variance.

**Supplemental Figure 3.** Forest plot comparing (a) time to eye opening; and (b) extubation time between remimazolam and control groups. CI, confidence interval; IV, inverse variance.

**Supplemental Figure 4.** Forest plot comparing the risk of postoperative nausea/vomiting (PONV) between remimazolam and control groups. M-H, Mantel-Haenszel; CI, confidence interval.

**Supplemental** **Table 1.** Search strategies for Medline

| Step | Key words or Mesh terms |
| --- | --- |
| 1 | ("General anesthesia*" or "Tracheal intubation" or "anesthetic induction" or "extubation" or "surgery" or "postoperative" or "intraoperaitve" or "perioperative" or "surgical patients" or "induction").mp. |
| 2 | exp "Anesthesia, General"/ |
| 3 | ("Remimazolam" or "CNS 7056").mp. |
| 4 | (1 or 2) and 3 |
| 5 | 4 and (((randomized controlled trial or controlled clinical trial).pt. or randomi*ed.ab. or placebo.ab. or drug therapy.fs. or randomly.ab. or trial.ab. or groups.ab.) not (exp animals/ not humans.sh.)) |

**Supplemental table 2:** Summary of findings for the main comparison

|  | | | | | | |
| --- | --- | --- | --- | --- | --- | --- |
| Outcomes | **Effect (Risk)** | | Relative effect (95% CI) | № of participants  (studies) | Certainty of the evidence (GRADE) | Comments |
|  | **Intervention group** | **Control group** |  |  |  |  |
| Risk of hypotension | 89/361 | 106/283 | **RR 0.6** (0.48 to 0.74) | 644 (6 RCTs) | ⨁⨁⨁⨁ High | - |
| Depth of anesthesia (BIS value) | - | - | **MD 9.26** (3.06 to 15.47) | 490 (5 RCTs) | ⨁⨁◯◯ Low | a,b |
| Induction efficacy | 198/201 | 118/118 | **RR 0.98** (0.9 to 1.06) | 319 (2 RCTs) | ⨁⨁◯◯ Low | a,b |
| Loss of consciousness | - | - | **MD 15.49** (6.53 to 24.46) | 331 (3 RCTs) | ⨁⨁◯◯ Low | a, b |
| Injection pain | 0/237 | 36/170 | **RR 0.03** (0.01 to 0.16) | 407 (3 RCTs) | ⨁⨁⨁⨁ High | - |
| Heart rate | - | - | **MD 4.26** (0.01 to 8.51) | 265 (4 RCTs) | ⨁⨁◯◯ Low | a, b |
| Mean blood pressure | - | - | **MD 5.79**  (-0.5 to 12.07) | 265  (4 RCTs) | ⨁⨁◯◯ Low | a, b |
| Time to eye opening | - | - | **MD -1.12**  (-7.38 to 5.14) | 405  (3 RCTs) | ⨁⨁◯◯ Low | a, b |
| Time to extubation | - | - | **MD -4.59**  (-12.31 to 3.13) | 485  (4 RCTs) | ⨁⨁◯◯ Low | a, b |
| Nausea/vomiting | 10/211 | 11/208 | **RR 0.82**  (0.26 to 2.6) | 419  (5 RCTs) | ⨁⨁⨁⨁ High | - |

**Comments:**

^a^wide 95% CI

^b^The I square is more than 50%.

**GRADE Working Group grades of evidence:
High certainty**: We are very confident that the true effect lies close to that of the estimate of the effect
**Moderate certainty**: We are moderately confident in the effect estimate: The true effect is likely to be close to the estimate of the effect, but there is a possibility that it is substantially different
**Low certainty**: Our confidence in the effect estimate is limited: The true effect may be substantially different from the estimate of the effect
**Very low certainty**: We have very little confidence in the effect estimate: The true effect is likely to be substantially different from the estimate of effect

**Supplemental figure 1.** Forest plot comparing heart rate between remimazolam and control groups. CI, confidence interval; IV, inverse variance.


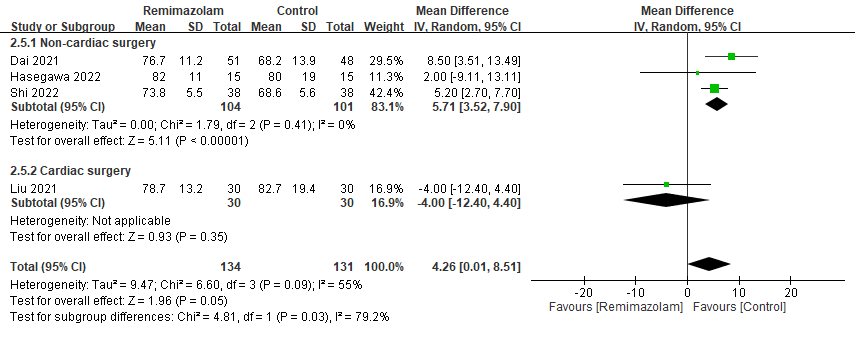


**Supplemental figure 2.** Forest plot comparing mean blood pressure between remimazolam and control groups. CI, confidence interval; IV, inverse variance.


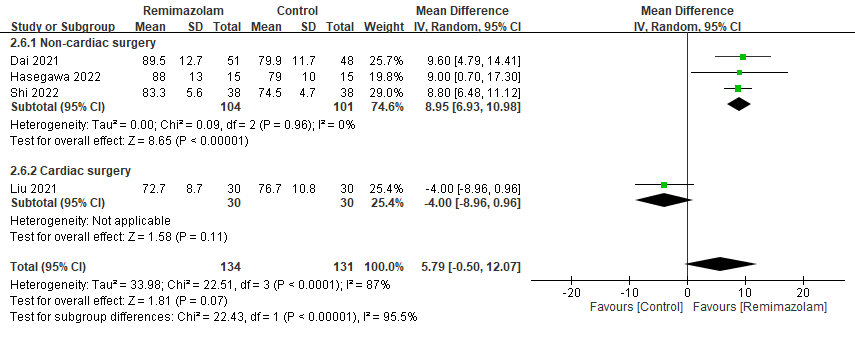


**Supplemental Figure 3.** Forest plot comparing (**a**) extubation time; and (**b**) time to eye opening between remimazolam and control groups. CI, confidence interval; IV, inverse variance.


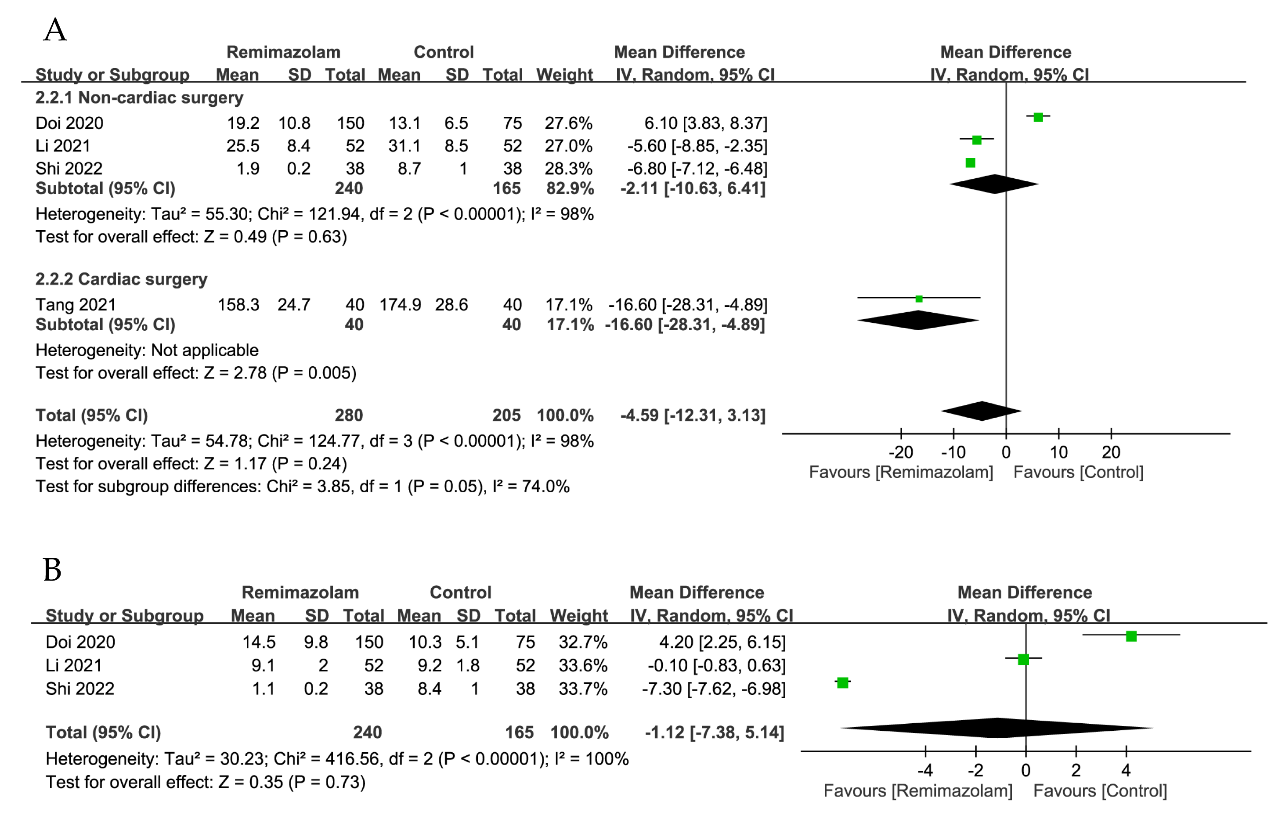


**Supplemental Figure 4.** Forest plot comparing the risk of postoperative nausea/vomiting (PONV) between remimazolam and control groups. M-H, Mantel-Haenszel; CI, confidence interval.


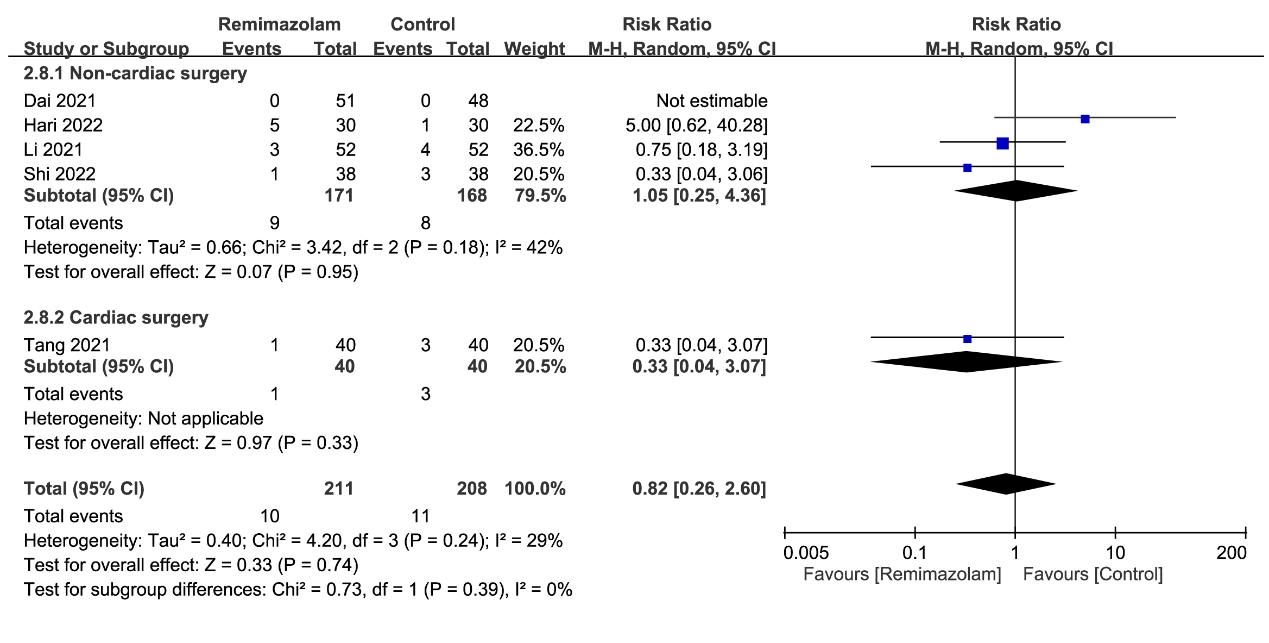

Supplement: Supplementary file 1 [file Table2.DOCX]
